# Growthcurver: an R package for obtaining interpretable metrics from microbial growth curves

**DOI:** 10.1186/s12859-016-1016-7

**Published:** 2016-04-19

**Authors:** Kathleen Sprouffske, Andreas Wagner

**Affiliations:** Department of Evolutionary Biology and Environmental Studies, University of Zurich, Winterthurerstrasse 190, Zurich, 8057 Switzerland; Swiss Institute of Bioinformatics, Quartier Sorge - Batiment Genopode, Lausanne, 1015 Switzerland; The Santa Fe Institute, 1399 Hyde Park Road, Santa Fe, New Mexico, 24105 USA

**Keywords:** Growth curve, Logistic, Experimental evolution

## Abstract

**Background:**

Plate readers can measure the growth curves of many microbial strains in a high-throughput fashion. The hundreds of absorbance readings collected simultaneously for hundreds of samples create technical hurdles for data analysis.

**Results:**

Growthcurver summarizes the growth characteristics of microbial growth curve experiments conducted in a plate reader. The data are fitted to a standard form of the logistic equation, and the parameters have clear interpretations on population-level characteristics, like doubling time, carrying capacity, and growth rate.

**Conclusions:**

Growthcurver is an easy-to-use R package available for installation from the Comprehensive R Archive Network (CRAN). The source code is available under the GNU General Public License and can be obtained from Github (Sprouffske K, Growthcurver sourcecode, 2016).

## Background

By tracking bacterial growth over time, important population-level information can be assessed, including doubling time and carrying capacity. Typical experiments entail measuring bacterial cell density at a series of time intervals, and then fitting these observations to an exponential growth model. Such measures can be made in parallel in a plate reader, and may result in hundreds or thousands of absorbance measurements over the course of 24 h. The resulting growth curves are commonly used in a variety of microbiological experiments [1–3], including experimental evolution [4–7]. A variety of methods have been used to obtain metrics from such growth curves [1, 3, 4, 8–13]. Older methods relied on manually plotting the cell count or absorbance readings over time on semi-log graph paper to obtain metrics like the maximum growth rate [8], an approach which has been computationally mirrored recently by GrowthRates [12]. Other recent methods fit the growth data to a variety of parametric growth models [11], but have a strong focus on generating dose response curves.

Here, we fit growth curve data to the standard form of the logistic equation common in ecology and evolution [14, 15] whose parameters (the growth rate, the initial population size, and the carrying capacity) provide meaningful population-level information with straight-forward biological interpretation. We implemented this as the R package Growthcurver, available for download at Comprehensive R Archive Network (CRAN), and provide a simple data analysis work-flow in the vignette.

## Implementation

We developed an open-source R package, Growthcurver, to obtain a variety of easily-interpretable metrics to summarize microbial growth curve data. Growthcurver is available from CRAN and the source code is available under the GNU General Public License.

### Carrying capacity and growth rate

Growthcurver fits a basic form of the logistic equation common in ecology and evolution [14, 15] to experimentally-obtained growth curve data. The logistic equation gives the number of cells *N*_*t*_ at time *t*.

$$ N_{t} = \frac{K}{1 + \left(\frac{K-N_{0}}{N_{0}} \right) e^{-rt}} $$

Here, the population size at the beginning of the growth curve is given by *N*_0_. The maximum possible population size in a particular environment, or the carrying capacity, is given by *K*. The intrinsic growth rate of the population, *r*, is the growth rate that would occur if there were no restrictions imposed on total population size. Growthcurver finds the best values of *K*, *r*, and *N*_0_ for the growth curve data using the implementation of the non-linear least-squares Levenberg-Marquardt algorithm [16] available in the minpack.lm R package [17]. The carrying capacity and growth rate values (*K* and *r*) are particularly useful for summarizing and comparing the growth dynamics of strains.

### Area under the curve

Growthcurver computes the area under the logistic curve, which provides a metric (the logistic AUC) that integrates information from the logistic parameters (*k*, *r*, and *N*_0_). We implemented this feature by evaluating the definite integral of the fitted logistic equation from time 0 to a user-defined time *t*. Growthcurver also computes an empirical AUC by summing the areas of the trapezoids made up by connecting consecutive data points of cell counts (or absorbance measurements) from time 0 to time *t*, similar to what was done by [18].

### Doubling time

The doubling time, also called the generation time, of a population is the time it takes for the number of individuals (or the absorbance reading) to double. Growthcurver computes the fastest doubling time *t*_*DT*_ possible for the population according to

$$ t_{DT} = \frac{\ln 2}{r} $$

[15], which occurs when the population has no restrictions on its growth (e.g., when it is far from the carrying capacity).

## Results and discussion

### Usage

Growthcurver can be used to compute metrics for growthcurves in two modes: individually for a single sample, or in batch mode for an entire plate of samples.

In both cases, Growthcurver requires just two vectors of data for each growth curve: time measurements and absorbance readings taken at those times. The time measurements’ unit determines the unit of the metrics returned by Growthcurver (e.g., if the input data are in minutes, then the doubling time returned by Growthcurver is in minutes and the growth rate is in minutes ^−1^). The absorbance of the media should be subtracted from the absorbance readings, and Growthcurver provides an option to do that automatically.

Example calls to Growthcurver can be found in the R documentation for the functions SummarizeGrowth and SummarizeGrowthByPlate, and extensive sample code for obtaining, checking, and interpreting growth curves is provided in the accompanying vignette [19].

### Correlation between metrics

To compare different metrics computed by Growthcurver, we measured 937 growth curves of 33 different *Escherichia coli* K12 strains in 200 *μ*L of Davis Minimal broth (Fluka 15758-500G-f) supplemented with 1 *μ*g/L glucose (‘DM1000’ media). For each measurement, we started a pre-culture in 2 mL DM1000 media and grew it at 37 °C with shaking for 24 h. We diluted the overnight culture 10000-fold, transferred it into 96-well plates (TPP 92096), and placed it in a plate reader (Tecan Infinite 200 Pro). We grew the resulting 96 populations at 37 °C with shaking for 24 h, and took optical density (OD) readings at a wavelength of 600 nm every 10 min. We removed the contribution of the media from the optical density by subtracting the minimum observed OD value for each growth curve from the rest of the values. Finally, we used Growthcurver to obtain the growth curve metrics for these populations.

**Fig. 1:**
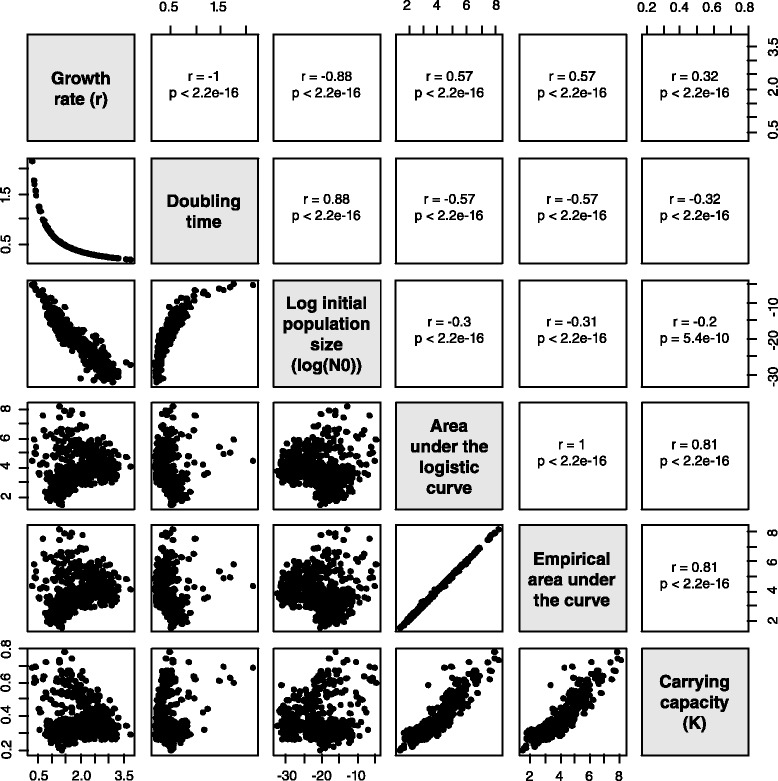
Comparisons of Growthcurver’s growth curve metrics for experimental data from 937 replicate *E. coli* populations. We plotted the growth curve metrics in a pairwise fashion to identify correlations between metrics. The metrics are listed in the diagonal (growth rate, doubling time, the logarithm of the initial population size, the area under the logistic curve, the area under the experimentally-measured curve, and the carrying capacity). We plotted the pairwise comparisons in the lower diagonal; for example, in the panel comparing growth rate and doubling time, each point is the growth rate and doubling time obtained from Growthcurver for a single experimental replicate. The Spearman correlation of any panel in the lower diagonal is reported in the upper diagonal

The growth rate is often used to summarize growth curve data, and we wondered to what extent the growth rate correlates with other metrics that summarize growth (Fig. 1). The growth rate correlates perfectly with the doubling time (Spearman, *ρ*=−1,*p*<2.2×10^−16^), which is unsurprising since the definition of doubling time relies on growth rate (see Implementation). The growth rate also correlates with the initial population size (Spearman, *ρ*=−0.88,*p*<2.2×10^−16^), the area under the curve (Spearman, *ρ*=0.57,*p*<2.2×10^−16^), and the carrying capacity (Spearman, *ρ*=0.32,*p*<2.2×10^−16^). The area under the curve is a promising metric to summarize a growth curve because it integrates the contributions of the initial population size, growth rate, and carrying capacity into a single value, and emphasizes growth rate (Spearman correlation between area under the curve and growth rate, *ρ*=0.57,*p*<2.2×10^−16^) and carrying capacity (Spearman correlation between area under the curve and carrying capacity, *ρ*=0.81,*p*<2.2×10^−16^). A viable non-parametric approach is to use the empirical area under the curve, which is correlated with the area under the curve (Spearman, *ρ*=1,*p*<2.2×10^−16^).

## Conclusions

Here, we have described the R package Growthcurver that provides several growth curve metrics with intuitive biological interpretation, including the growth rate, the doubling time, the carrying capacity, and the area under the logistic growth curve which integrates the contributions of the other metrics into a single value.

Growthcurver is available for installation from CRAN [20] or Github [21]. The accompanying vignette [19] provides step-by-step examples for analyzing a single sample and a plate of samples, as well as information on preparing the input data for analysis. Novice R users should be able to use the example code provided with few or no changes to analyze their own growth curve data.

The high-throughput nature of obtaining replicate growth curves in plates allows us to easily characterize hundreds of growth curves, even though caution is needed to interpret these metrics in terms of their effects on competitive fitness [22]. Growthcurver allows for the straightforward analysis and interpretation of growth curve data collected in a high-throughput manner using plate readers.

## Availability and requirements

- **Project name:** Growthcurver
- **Project home page:** http://github.com/sprouffske/growthcurver
- **Operating system:** Platform independent
- **Programming language:** R (version 3.2.2)
- **Other requirements:** The R Project for Statistical Computing
- **License:** GNU General Public License
- **Any restrictions to use by non-academics:** According to GNU General Public License

## Abbreviations

AUC: area under the curve
CRAN: comprehensive R archive network
